# Transthoracic robot-assisted minimally invasive diaphragmatic rupture repair: a case report

**DOI:** 10.1186/s13256-026-05944-w

**Published:** 2026-03-15

**Authors:** Raffaella Griffo, Alessio Campisi, Henrike Deissner, Benedikt Niedermaier, Peter Reimer, Hauke Winter, Martin E. Eichhorn

**Affiliations:** 1 Department of Thoracic Surgery, Thoraxklinik, Heidelberg University Hospital, Roentgenstraße 1, 69126 Heidelberg, Germany; 2 Thoracic Surgery Unit, Cardiovascular and Thoracic Department, University and Hospital Trust-Ospedale Borgo Trento, Verona, Italy; 3 Member of the German Center for Lung Research (DZL), Translational Lung Research Center Heidelberg (TLRC-H), Heidelberg, Germany; 4 Division of Systems Biology of Signal Transduction, German Cancer Research Center (DKFZ), DKFZ-ZMBH Alliance, Heidelberg, Germany

**Keywords:** Diaphragmatic rupture, Diaphragmatic plication, Robot-assisted thoracoscopic surgery, Minimally invasive surgery, Case report

## Abstract

**Background:**

Nontraumatic diaphragmatic rupture is a rare condition with clinical presentations ranging from asymptomatic to severe respiratory or gastrointestinal symptoms. Owing to its nonspecific nature, it can be difficult to differentiate from other diaphragmatic disorders.

**Case presentation:**

We present a case of a 35-year-old German woman with a right-sided nontraumatic diaphragmatic rupture, presenting with recurrent hypertensive crises and acute dyspnea. Although initially misdiagnosed, further clinical and radiological evaluations suggestive of diaphragmatic elevation led to the decision to proceed with robot-assisted thoracoscopic surgery.

**Conclusion:**

This case underscores the diagnostic challenges of non-traumatic diaphragmatic rupture, especially in complex clinical scenarios. It highlights the importance of thorough clinical assessment, appropriate imaging, and timely surgical intervention. Robot-assisted thoracoscopic surgery proved to be a safe and effective therapeutic option and should be considered in select cases where conventional diagnostics fail to provide definitive answers.

## Introduction

Non-traumatic diaphragmatic rupture is a rare condition with a varied and often nonspecific presentation, making early diagnosis challenging. Its subtle clinical signs are frequently mistaken for other diaphragmatic disorders, and preoperative diagnosis is difficult, often misidentified as diaphragmatic elevation. Such errors can delay appropriate surgical intervention and compromise patient outcomes. We present a unique case of non-traumatic diaphragmatic rupture successfully repaired using a robot-assisted minimally invasive approach, with the concomitant presence of a pheochromocytoma whose hypertensive effects were initially masked and only uncovered following treatment of the diaphragmatic defect.

## Case presentation

A 35-year-old German woman was admitted to our hospital with right diaphragmatic elevation. Arterial hypertension was the only comorbidity reported. Her medical history was notable for recurrent episodes of acute dyspnea, tachycardia, and hypertensive crises, which progressively worsened following a full-term pregnancy with spontaneous vaginal delivery 2 years earlier. Owing to the disabling symptoms, the patient reported significant physical suffering and consequent psychological distress, with a marked deterioration in her quality of life. She denied any history of trauma. There was no known family history of diaphragmatic disorders or tumors.

The physical examination revealed no pathological findings, and blood test results were within normal limits. Pulmonary function testing demonstrated a mild restrictive ventilatory defect.

**Fig. 1:**
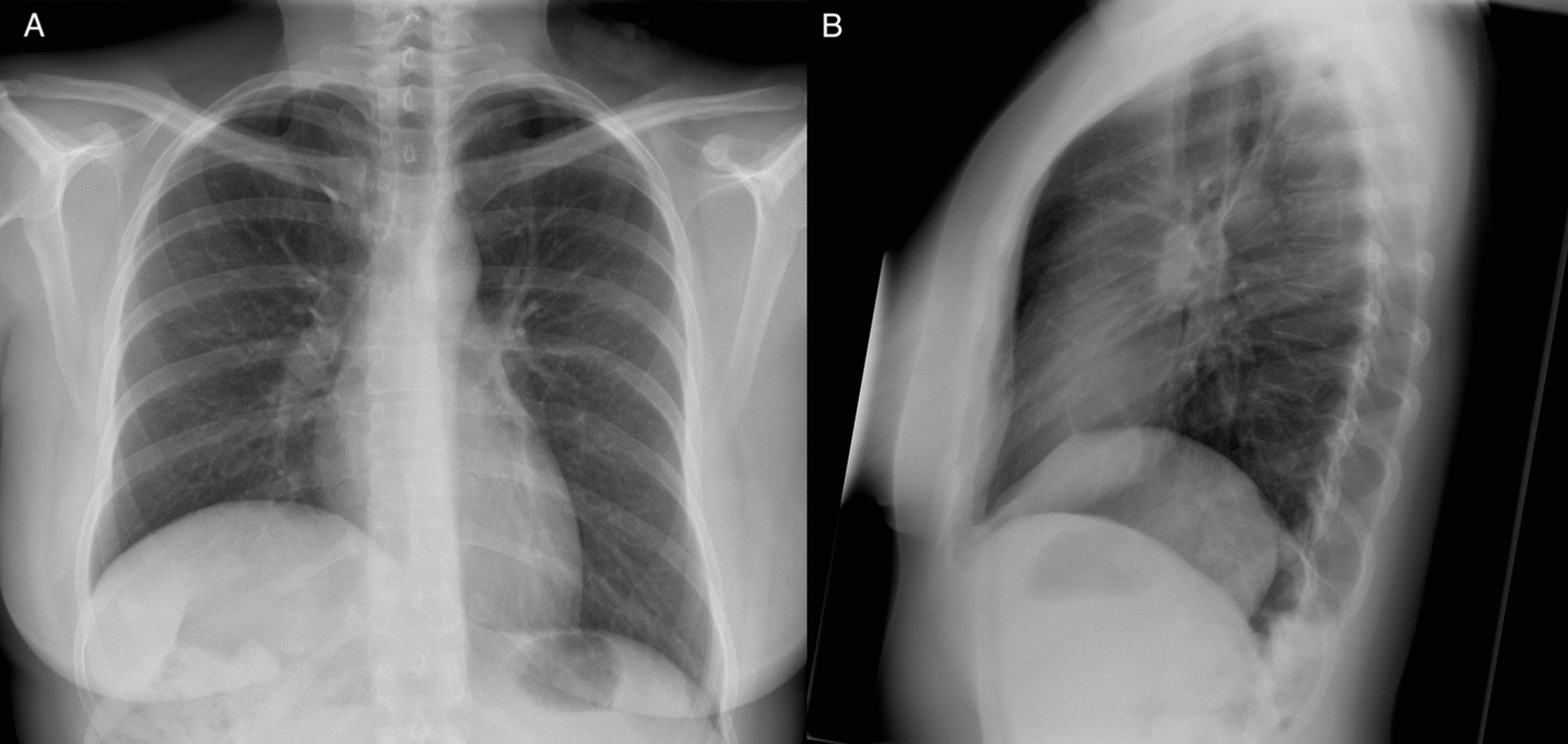
Posteroanterior (**a**) and lateral (**b**) chest X-rays demonstrating significant right-sided diaphragmatic elevation

**Fig. 2:**
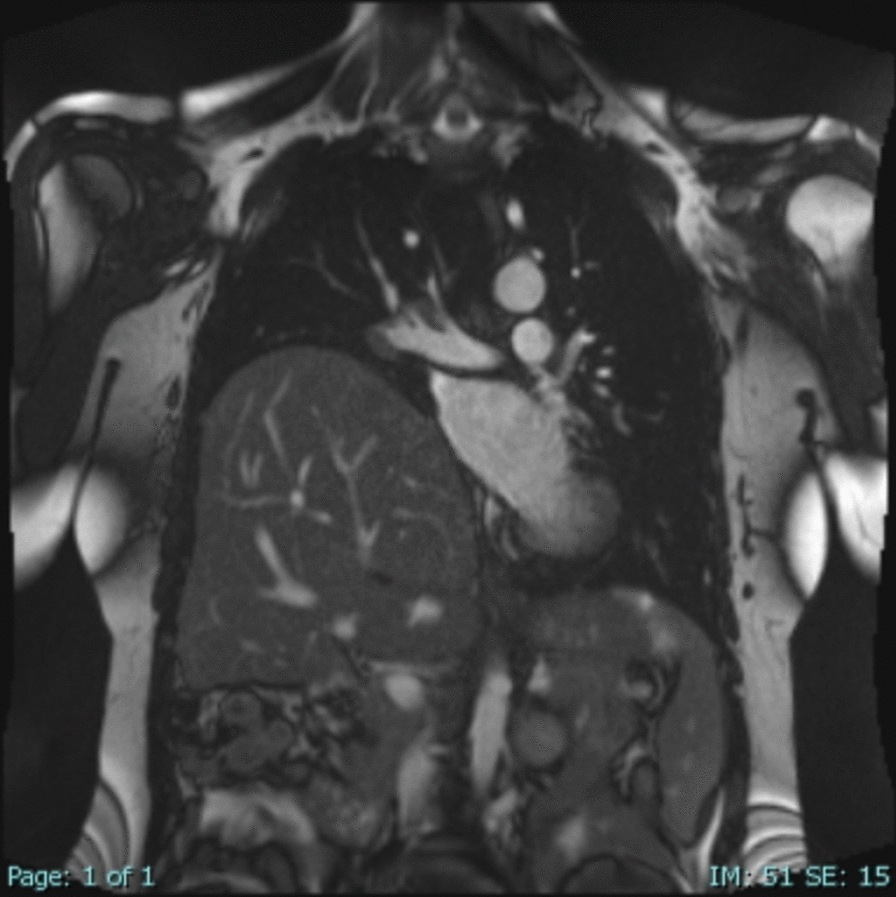
Preoperative dynamic sagittal chest magnetic resonance imaging showing a significantly elevated right hemidiaphragm with reduced mobility

**Fig. 3:**
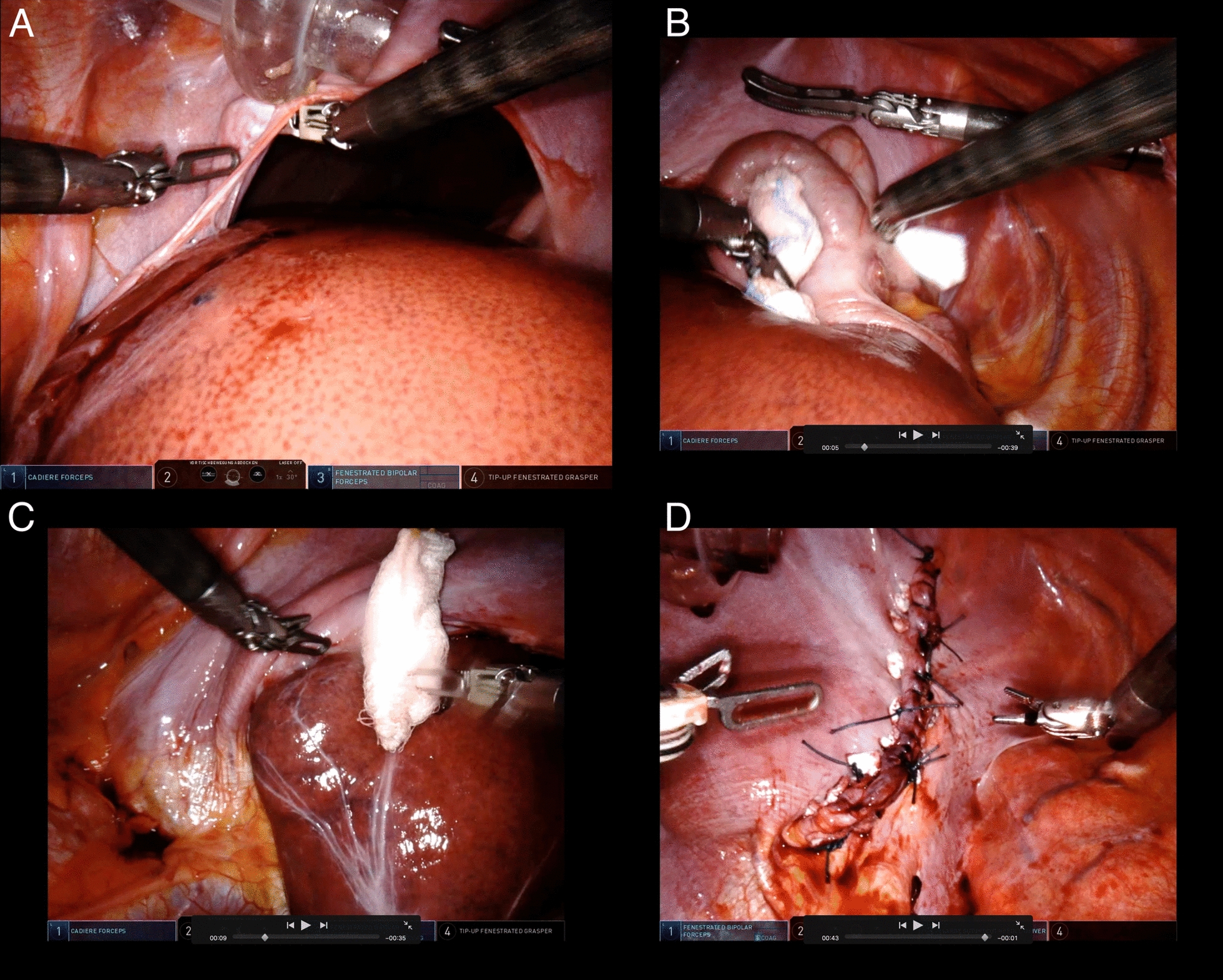
Intraoperative view revealing herniation of the liver and colon through a wide central tendon rupture of the right hemidiaphragm (**a**). Repositioning of the colon (**b**) and the liver into the abdominal cavity (**c**), and defect repair using polytetrafluoroethylene (PTFE)-pledget-reinforced Ethibond non-resorbable sutures (**d**)

A chest X-ray revealed right diaphragmatic elevation (Fig. 1). Following the exclusion of cardiac etiologies for her symptoms, the patient was referred for comprehensive evaluation of suspected diaphragmatic dysfunction. Dynamic magnetic resonance imaging (MRI) confirmed an elevated right hemidiaphragm (Fig. 2), but neither diaphragmatic paralysis nor rupture was detected. Functional imaging with fluoroscopy showed paradoxical movement of the right hemidiaphragm, indicating reduced motility without complete paralysis. Given the patient’s young age, persistent symptoms despite physiotherapy, and interdisciplinary discussions, we opted for an exploratory right-sided robot-assisted thoracoscopic surgery (RATS). A full portal robotic four-arm approach using the DaVinci Xi-System was performed. The patient was positioned in the left lateral decubitus position with one-lung ventilation. After the placement of three 8-mm trocars and a utility port, CO_2_ was insufflated. Intraoperatively, a large diaphragmatic rupture was unexpectedly discovered. The liver and intestines had herniated into the thoracic cavity, likely causing vena cava inferior compression syndrome. To mobilize and reposition the abdominal organs, a fourth 8 mm robotic port was inserted. The margins of the diaphragmatic defect were mobilized, and the diaphragm was repaired using polytetrafluoroethylene (PTFE)-pledget-reinforced non-resorbable sutures (Ethibond Excel™ 2, Ethicon, Norderstedt, Germany) (Fig. 3). Additional reinforcement with an alloplastic mesh was avoided to preserve the diaphragm’s contractility. No significant intraoperative blood loss was observed. The surgical procedure lasted a total of 123 minutes.

**Fig. 4:**
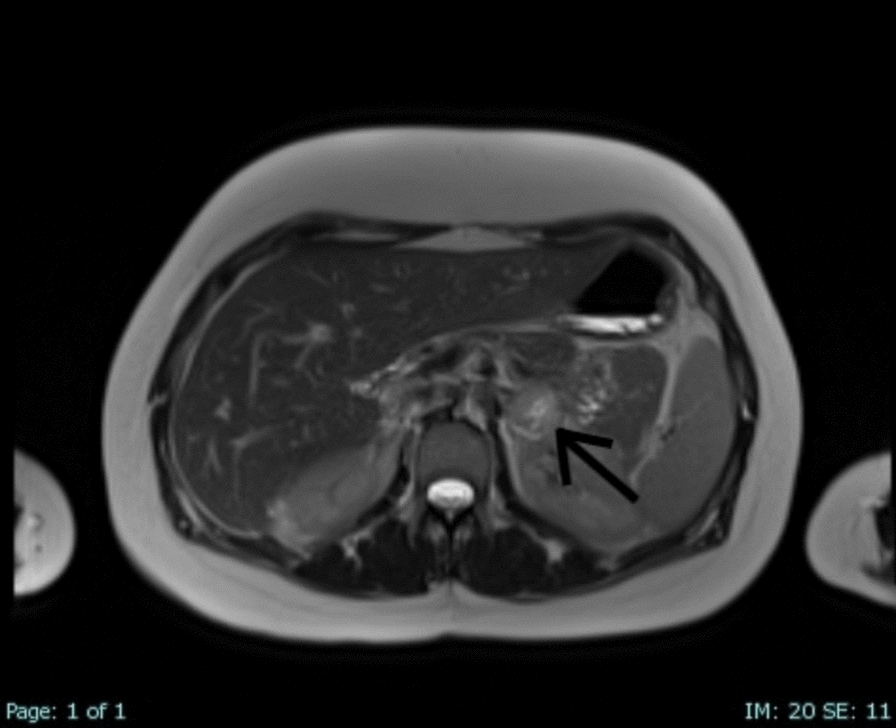
Postoperative dynamic abdominal coronal magnetic resonance imaging showing a tumor lesion in the left adrenal gland, indicated by the black arrow.

**Fig. 5:**
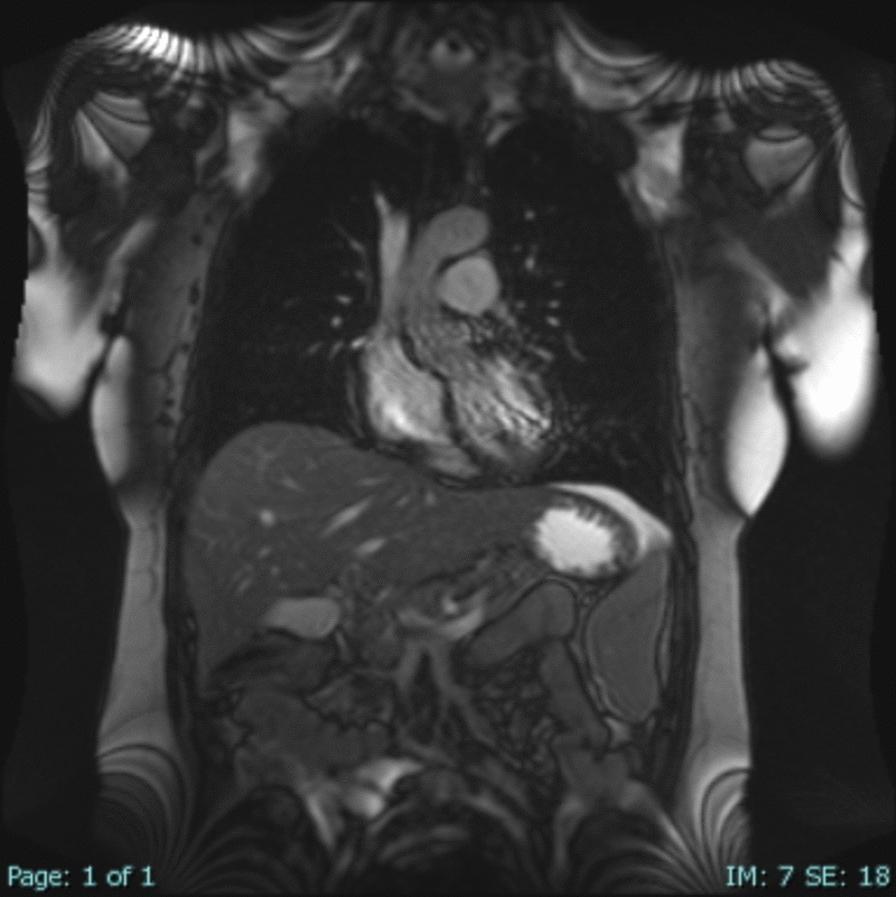
Postoperative dynamic sagittal chest magnetic resonance imaging demonstrating a symmetrical diaphragm on both sides, without evidence of elevation or re-rupture on the right side

The patient’s postoperative recovery was uneventful. Imaging confirmed significant improvement in right lung expansion and resolution of respiratory symptoms. However, 10 days after discharge, the patient presented again with a hypertensive crisis. Laboratory tests were unremarkable, and chest X-ray findings were normal, showing proper alignment of the right diaphragm. Further investigation with dynamic chest and abdominal MRI revealed a contrast-enhancing tumor mass in the left adrenal gland measuring 2.3 cm in diameter (Fig. 4), which was not diagnosed on preoperative dynamic MRI imaging. The case was presented to a multidisciplinary team, including endocrinologists and general surgeons. Given the clinical suspicion of pheochromocytoma, targeted biochemical testing was performed, revealing markedly elevated plasma levels of metanephrine, normetanephrine, norepinephrine, epinephrine, and dopamine. Urinary analysis also demonstrated significantly increased levels of norepinephrine and vanillylmandelic acid. On the basis of these findings, oral antihypertensive therapy was initiated to optimize the patient’s condition prior to surgery, and laparoscopic adrenalectomy was scheduled. The postoperative course was uneventful, with no complications observed. Histopathological analysis confirmed the diagnosis of pheochromocytoma without capsular invasion, and no adjuvant treatment was warranted. The patient underwent regular oncological follow-up with no evidence of tumor recurrence. In addition, she was monitored surgically at our center with follow-up evaluations at 6, 12, and 24 months postoperatively. Dynamic MRI scans performed at 1 and 2 years after thoracic surgery demonstrated normal bilateral diaphragmatic motion, with no evidence of diaphragmatic elevation or dysfunction. (Fig. 5).

## Discussion

This case reflects the complex interplay between two coexisting conditions: a chronic diaphragmatic rupture and an undiagnosed pheochromocytoma. While the hypertensive crises were clearly attributable to the pheochromocytoma, our hypothesis is that the diaphragmatic rupture significantly contributed to the respiratory symptoms and, in fact, played a pivotal role in prompting further investigations. Without the presence of radiological and clinical signs of diaphragmatic dysfunction, the adrenal mass might have remained undetected until more severe systemic consequences emerged. Comparable cases have not been previously documented in literature. Although there are isolated reports of pheochromocytomas located near or involving the diaphragm, the coexistence of a chronic diaphragmatic rupture and a pheochromocytoma has not been described before, making this a uniquely complex and unprecedented clinical scenario [1–3].

An additional noteworthy aspect is that, although the diaphragmatic rupture was chronic, likely dating back approximately 2 years, the nonspecific nature of the patient’s symptoms significantly hindered both the diagnostic process and the initial clinical management. Such diagnostic uncertainty is frequently encountered in diaphragmatic pathologies. However, the persistence and gradual worsening of symptoms ultimately prompted a more comprehensive evaluation, which led to the correct diagnosis [4]. At our center, surgical treatment of diaphragmatic elevation is considered only after a comprehensive evaluation of the underlying pathology and symptom burden. Surgery is reserved for cases where diaphragmatic paralysis with paradoxical movement is documented; otherwise, a conservative approach is preferred. Although complete diaphragmatic paralysis was never observed in our patient, the choice to pursue an individualized, unconventional strategy underscores the critical importance of personalized management in diaphragmatic disorders, especially in cases presenting with overlapping or ambiguous clinical symptoms [4, 5].

Furthermore, the use of a transthoracic robot-assisted approach demonstrated high efficacy. It allowed for detailed inspection of the pleural cavity, safe lysis of adhesions, meticulous bleeding control, and a tension-free repair of the rupture. Intraoperative CO_2_ insufflation was particularly helpful in reducing intra-abdominal pressure and promoting spontaneous repositioning of abdominal organs, avoiding the need for forced traction [5–7]. Finally, a suture-only repair technique was employed, avoiding the use of synthetic mesh and thus preserving the diaphragm’s physiological mobility. This was confirmed by follow-up dynamic MRI studies at 12 and 24 months, which showed excellent bilateral diaphragmatic excursion and no evidence of recurrence.

## Conclusion

This case illustrates the diagnostic complexity that can arise from the coexistence of multiple pathologies with overlapping symptoms. While the hypertensive crises were ultimately linked to an adrenal pheochromocytoma, it was the diaphragmatic rupture that brought the patient to medical attention. The combination of clinical vigilance, advanced imaging, and interdisciplinary collaboration enabled timely diagnosis and treatment of both conditions. On the basis of our experience, robot-assisted transthoracic repair represents a safe, effective, and minimally invasive option for managing chronic diaphragmatic rupture. This approach allows for accurate identification and repair of the defect, minimizes perioperative trauma, and facilitates preservation of diaphragmatic mobility, an essential factor for long-term respiratory function. Careful patient selection and individualized surgical planning remain key to achieving optimal outcomes in such complex scenarios.

## Data Availability

The data that support the findings of this study are available from the corresponding author upon reasonable request.

## Abbreviations

MRI: Magnetic resonance imaging
RATS: Robot-assisted thoracoscopic surgery
PTFE: Polytetrafluoroethylene
